# Emotion Regulation in Families: Exploring the Link between Parent-Child Alexithymia and Child Post-Traumatic Stress Symptoms Amidst the COVID-19 Pandemic

**DOI:** 10.1007/s10578-024-01728-9

**Published:** 2024-06-14

**Authors:** Casey B. Corso, Rebecca Hoppe, Wendy Kliewer, Traci Wike, Marcia A. Winter

**Affiliations:** 1 Department of Psychology, Virginia Commonwealth University, 808 West Franklin St, Box 842018, Richmond, VA 23284-2018 USA; 2 School of Social Work, Virginia Commonwealth University, Box 842027, Richmond, VA 23284-2027 USA

**Keywords:** Alexithymia, Posttraumatic stress, COVID-19, Emotion regulation, Trauma

## Abstract

The COVID-19 pandemic comprises a mass trauma for children and families, and children may face particular vulnerability to post-traumatic stress symptoms (PTSS) through processes of parent and child emotional dysregulation, such as alexithymia. With 88 U.S. children (*M*_age_ = 9.94 years; 54.5% female; 59.1% White) and their parents/caregivers (68.2% female; 59.1% White), a path model was tested in which child alexithymia symptoms partially mediated the association between parent alexithymia symptoms and child COVID-19-related posttraumatic stress symptoms (PTSS). We also tested an alternative model in which child alexithymia symptoms moderated the association between parent alexithymia symptoms and child PTSS. The hypothesized mediation model was supported (β = 0.15, *SE* = 0.05, 95% CI: [0.07, 0.25], *p* < 0.001), whereas the hypothesized moderator model was not (β = 0.06, *p* = 0.44). Findings highlight the importance of parents’ emotional understanding and regulation for child mental health during mass traumas such as pandemics.

## Clinical Trial Registration

This study was not a part of a clinical trial, and thus not applicable for clinical trial registration.

Like war or natural disasters, the COVID-19 pandemic can be considered a mass trauma (i.e., an adverse event which occurs simultaneously across large groups of individuals) [1]. As a mass trauma, COVID-19 poses direct risk to individuals’ health and well-being and undermines protective systems usually afforded to children, such as parent-child relationships, family systems, school systems, childcare facilities, and community connections. Thus, COVID-19 provides a unique milieu in which to examine risk and protective factors linked to stress and mental health outcomes. Such research can inform interventions that incorporate the child’s contributions to these processes along with proximal relational and social factors that can mitigate negative outcomes for children [2].

## Posttraumatic Stress after Mass Traumas

Post-traumatic stress disorder (PTSD) is considered the most common psychopathological outcome following mass trauma [3–5]. Consequences of PTSD in children are profound and can include substantial impairment in social functioning, academic performance, and subsequent mental health [6, 7]. Beyond diagnosed PTSD, posttraumatic stress symptoms (PTSS) indicate a complex trauma response [8]. PTSS are long-lasting after mass traumas for both adults and children [9, 10]. Children with PTSS are at risk for impairments in social, emotional, behavioral, cognitive, and physical domains [8, 11]. Moreover, while direct exposure to traumatic events is strongly associated with PTSD, PTSS is more likely to be experienced following indirect exposure to traumatic events, such as when an event happens to a loved one [12]. In the context of COVID-19, PTSS may be a particularly salient construct for study given children’s direct and indirect trauma exposures.

Emerging COVID-19 literature shows that post-traumatic stress (PTS), marked by PTSD and/or PTSS, is a concern for children and families [13, 14]. COVID-19 presents unprecedented risk to mental health globally, with PTSD ranging in prevalence from seven to 53.8% across eight countries [15]. In a sample of 1,040 adult participants across five Western countries, PTSS was found to exist among a variety of exposure types (e.g., direct and indirect exposure, anticipated and unanticipated events) and were best predicted by self-reported emotional impact of the events [16]. Further, researchers have concluded that COVID-19 should be understood as a traumatic stressor with potential to lead to PTSD diagnosis in those ages 16 years and older [14, 17–19]. In younger children, research also reveals the potency of mass traumas in that they commonly result in heightened levels of PTSS and PTSD [20, 21]. Moreover, child self-reports have shown higher rates of symptoms than parent-reports of child PTSS during COVID-19 [21], indicating the importance of assessing children’s PTSS from children themselves.

A recent systematic review further evidenced the impact of COVID-19 on children and families by conceptualizing the COVID-19 pandemic and its containment measures as traumatic events; the review evaluated 22 primary studies and found that the pandemic and its containment measures were linked to increased trauma-related symptoms, including PTSS, depression, and anxiety, in both children and caregivers [22]. For instance, Davico et al. (2021) found that 30.9% of children assessed were at high risk for PTSD [23], while Ma et al. (2021) reported a PTSD prevalence of 20.66% among their sample [24]. It is also noted in this review that children’s trauma symptoms are often correlated with their caregiver’s psychological distress, emphasizing the interconnected nature of family mental health during the pandemic.

## Intergenerational Transmission of Alexithymia (Fig. 1, Path a)

In the wake of a mass trauma, exploring factors that contribute to the emotional climate of the parent-child relationship as well as the psychological sequelae that may stem from those factors is critical to understanding how heterogeneous stress outcomes are developed among youth. Emotion regulation is the functional ability to manage emotional experiences and expressions via awareness, understanding, and acceptance of emotions in order to meet goals or demands of a situation [25–28]. Difficulty in regulating emotion is an underlying factor in nearly all clinical disorders and has been specifically linked to heightened PTSS [29, 30]. In the current study we examined emotion dysregulation through the more specific construct of alexithymia.

Emotion dysregulation often is used as an umbrella term [29], referring broadly to an individual’s emotional behavior and coping strategies. In contrast, alexithymia specifically refers to an individual’s difficulty or inability to recognize, describe, and communicate one’s emotions accurately. Alexithymia may be associated with difficulty in differentiating feelings from other bodily sensations [31, 32]. Alexithymia can be transmitted intergenerationally from parent to child, influencing child attitudes, traits, and behaviors. For example, parental alexithymia scores have been associated with children’s emotional difficulties, higher risk of psychopathology, higher alexithymia levels [33–35], emotionally restrictive home environments, and poor emotional communication [35, 36].

Theoretically, alexithymia may be transmitted from parents to children through social learning as well as direct teaching and socialization. Children mirror parent alexithymia characteristics [33, 36], and emotion socialization practices contribute directly to the intergenerational transmission of alexithymia [36–38]. Emotion socialization is influenced by implicit and explicit parental interaction with children around emotional topics [39–41]. Parent-child interactions such as those in the attachment system provide contexts for children to learn how to regulate emotions. When children feel uncertain or threatened, they look to the parent for interpretation and protection; how attuned to the children’s signals the caregiver is influences the sensitivity of their reaction. For example, caregivers’ sensitivity and responsiveness to children’s emotions are associated with adolescents’ emotion regulation [42].

From an attachment theory perspective, emotional understanding and regulation may also be conveyed to children via mentalizing, defined as the understanding of one’s own and others’ internal mental states (including emotions) [43]. Parental mentalizing, or parents’ capacity to represent and remain mindful of their children’s internal states [44] influences the quality of caregiving and children’s socio-emotional development [45]. Alexithymia is associated with poorer mentalizing [46].

**Fig. 1:**
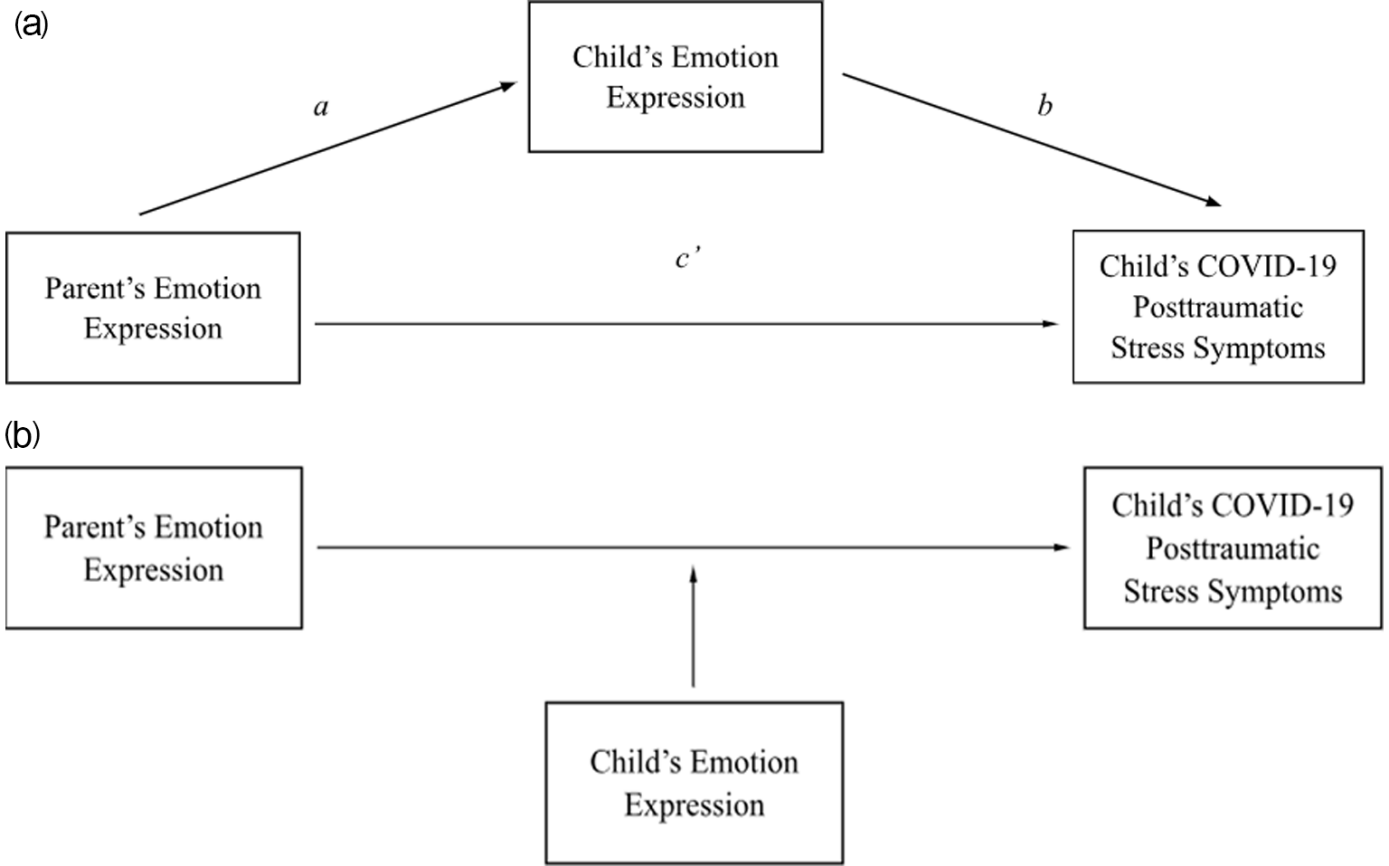
(**a**) Mediator model (**b**) Moderator model

Similarly, parental meta-emotion philosophy – overarching concepts regarding parents’ views of their own and others’ emotions – posits that meta-emotion styles taught by parents could strongly influence children’s emotion regulation development [47]. Meta-emotion philosophy is conceptualized as a parenting dimension, such that parents’ meta-emotion styles influence their behaviors around emotion awareness, acceptance, and coaching; parents’ emotion beliefs regarding themselves also link to their emotion beliefs regarding their children [40]. Research indicates that meta-emotion philosophy mediates the relationship between emotion coaching and children’s social competence [48] and that better parent emotion coaching skills are associated with higher child emotional competencies [49]. This corroborates findings from research on parent modeling and socialization, which has linked parental alexithymia to child outcomes such as lack of emotional and social competence [48–50]. Thus, the current study proposes a pathway (Fig. 1, section a, Path a-b) in which parent’s difficulties in identifying, understanding, and expressing their emotions leads to their children experiencing those same difficulties (Fig. 1, section a, Path a) which then influence the child’s interpretation of and subsequent stress related to COVID-19 (Fig. 1, section a, Path b).

### The Association between Child Alexithymia and Child Stress Outcomes (Fig. 1, Path b)

A child’s ability to identify, understand, and discuss their emotion leads them to possess adequate skill sets to control their arousal during stressful events. This association has been consistently evidenced in the literature. For example, toddlers who communicated their feelings were less likely to become distressed during stressful situations [51] and youth’s emotional regulation and reactivity has been linked to parental emotion-related socialization behaviors [52]. However, a meta-analysis noted that the inverse of this also was evidenced: non-supportive emotion socialization behaviors were associated with child emotion regulation difficulties and more internalizing psychopathology [53]. Indeed, children who are unable to freely discuss their emotions may be disadvantaged in their social and emotional competence due to their lack of expression and understanding [39]. Furthermore, alexithymia symptoms within families have been linked with general family pathology [54]. The data raise concerns about the influence of parental alexithymia in the context of COVID-19 on families and children. Indeed, individual differences in PTS outcomes during previous mass traumas have been linked with alexithymia tendencies [55–57]. Emerging COVID-19 literature supports this notion, where increased report of alexithymia symptoms was associated with psychopathology such as depression and PTSD [58, 59], with researchers concluding that alexithymia is a risk factor for developing psychopathology related to COVID-19 [60, 61]. Such findings indicate what factors may be contributing to heterogeneous outcomes, with alexithymia tendencies being a specific vulnerability [58–61].

Mass trauma literature indicates that parental displays of emotional distress following a stressful event are linked to higher levels of PTS in children [62]; terrified parents can terrify children [2, 63]. Emerging COVID-19 research supports this finding, such that children of parents with high levels of emotion coaching had lower levels of pandemic-related stress, whereas children of parents who reported higher levels of stress and pandemic-related anxiety had higher levels of pandemic-related stress [64]. Likewise, caregiver pandemic-related distress is positively related to children’s mental health problems [65]. Parental mental health problems exacerbated the negative effects of the pandemic in families and children [65–67], and families with more negative interactions had more difficulty adjusting to COVID-19 [68, 69]. Thus, research is needed that examines how alexithymia symptomatology and behaviors may be transmitted intergenerationally and affect child COVID-19 PTS outcomes. Where parenting via emotion socialization and meta-emotion philosophy occurs, parents with alexithymia symptoms may struggle to effectively socialize their children to use adaptive emotional responses and coping styles regarding the pandemic and associated feelings of stress.

Of particular interest is how these heterogeneous stress outcomes may develop during middle childhood (ages 8–12 years). Middle childhood, where children transition into adolescence, is considered a critical developmental period across a variety of domains (e.g., physical, neurological, cognitive, social, emotional) [70–73]. Middle childhood functioning is a more accurate predictor of long-term adjustment and success than other developmental periods [71] and middle childhood is considered a period of increased risk for long-term psychopathology [71, 74]. Further, pubertal onset during middle childhood is a sensitive period for trauma reactivity [75]. Thus, experiencing trauma during this period may have a particularly negative effect on development [72]. Moreover, children in middle childhood are building autonomy and increasingly needing to regulate their emotions independently from caregivers, yet they remain reliant on parents’ availability and guidance during emotionally-taxing events [76]. Therefore, in the wake of a mass trauma, exploring factors that inform the emotion climate of the dyad – such as alexithymia symptoms – and the subsequent psychological responses that may stem from those factors, is critical to understanding how heterogeneous stress outcomes are developed among youth.

### The Current Study

The current study examined the direct and indirect paths between parents’ emotion expression abilities and children’s stress response to COVID-19. Parents with alexithymia symptoms may struggle to effectively socialize their children to express emotions and cope in ways that contribute to better understanding of the pandemic and associated feelings of stress. First, we hypothesized that parents’ emotional expression challenges (i.e., greater difficulty identifying feelings and difficulty describing feelings) would be positively associated with their children’s COVID-19-related PTSS (Fig. 1, section a, Path c’). Second, we explored whether the association between parent alexithymia and child PTSS would be partially explained by children’s emotional expression abilities, such that parents’ greater difficulty identifying and describing feelings would be associated with poorer emotional awareness and more expressive reluctance in children, which in turn would be associated with greater child PTSS.

It may be the case that child emotional expression is only associated with PTSS when parental problematic emotional expression is high. That is, parental difficulties in emotional expression may exacerbate the association between child emotional expression and PTSS. Low parental problematic emotional expression may be protective for children. A moderator model allows one to test these hypotheses, thereby assessing when or for whom child emotional expression is associated with PTSS. Therefore, we also tested whether child emotion moderated the association between parent emotion and child stress (see Fig. 1, section b), acknowledging the complex interplay of factors influencing children’s emotion regulation skills.One advantage to such an argument is the lack of dependency child alexithymia has on parent alexithymia. Where children experience a multitude of proximal influences (e.g., school, peers, caregivers) all of which may influence their emotion regulation skills, it could be advantageous to understand child alexithymia as an influence on the existing proximal relations rather than an explanatory process, leading to flexibility in understanding child outcomes as they are informed by the dyad.

## Method

### Participants and Procedures

Participants in the current study were drawn from the Families Adapting to COVID-19 (FACT) study, which was approved by the Institutional Review Board at Virginia Commonwealth University. The cross-sectional study targeted English-speaking U. S. children ages 8–12 and their primary caregivers. FACT involved an online survey, administered via Qualtrics and social media outlets, focusing on family functioning following COVID-19. After the eligibility screening and completion of the parent survey, families were compensated with a $25.00 electronic gift card. Parents were then offered the opportunity to have their child participate in an additional survey. Both parent consent and child assent were obtained prior to survey administration. For this study, only participants with complete data on the variables of interest were included.

Families in the current study (*N* = 88) consisted of children ages 8 to 12 years (*M* = 9.94, *SD* = 1.33; 54.5% female) and their primary caregiver ages 25 to 57 years (*M* = 36.23, *SD* = 6.19; 68.2% female). Per parent report, the majority of children (*n* = 52, 59.1%) were White; 14 (15.9%) were Black or African American, 10 (11.4%) Hispanic or Latinx, 6 (6.4%) multi-racial, 3 (3.4%) Asian, 2 (2.3%) American Indian or Alaskan Native, and 1 (1.1%) Native Hawaiian or other Pacific Islander. Fifty-two (59.1%) primary caregivers self-identified as White, 13 (14.8%) as Black or African American, 10 (11.4%) Hispanic or Latinx, four (4.5%) multi-racial, five (5.7%) Asian, three (3.4%) American Indian or Alaskan Native, and one (1.1%) Native Hawaiian or other Pacific Islander. Most primary caregivers were college educated: 12 (13.6%) reported receiving a high school diploma/GED or less, 37 (42.0%) attended some college, and 39 (44.3%) obtained a college degree. In terms of household income, nine (10.3%) earned $0–9,999, 32 (36.3%) earned $10,000–39,999, 20 (22.5%) earned $40,000–84,999, and 27 (30.5%) earned $85,000-164,999).

### Measures

#### Parental Comfort with Emotion

The Toronto Alexithymia Scale-20 (TAS-20) [77] is a 20-item self-report measure for assessing comfort with emotion and helping to illuminate how parents socialize their children to cope. Items rated on a Likert scale of 1 (*Not at all like me* or *Not true*) to 5 (*Completely like me* or *Very true*) are summed into subscales. This study used the difficulty identifying feelings (DIF; e.g., “I am often confused about what emotion I am feeling”) and difficulty describing feelings (DDF; e.g., “It is difficult for me to reveal my innermost feelings, even to close friends”) subscales. Psychometrics in previous samples have been acceptable, with test-retest reliability reported as *r* = 0.71 (DIF) and *r* = 0.68 (DDF) [78] and internal consistency reported as α = 0.78–0.81 (DIF) and α = 0.75 (DDF) [77]. Based on the high correlation of DIF and DDF scores in the current study (*r*(86) = 0.70, *p* < 0.001), we summed items from the DIF and DDF subscales to yield a total score in which higher scores indicate greater difficulty with identifying and describing feelings (α = 0.89).

#### Emotion Expression Difficulties in Children

The Emotion Expression Scale for Children (EESC) [79] is a 16-item self-report measure of emotion expression difficulties. Children rate items on a Likert scale from 1 (*Not at all true*) to 5 (*Extremely true*) where higher scores indicate poorer emotional functioning. The EESC consists of two 8-item subscales: poor emotion awareness (PA; e.g., “I have feelings that I can’t figure out”) and reluctance to express emotion (ER; e.g., “It is hard for me to show how I feel about somebody”). High internal consistencies for the two subscales (α = 0.83, PA; α = 0.81, ER) and moderate test-retest reliability (*r* = 0.59, PA; *r* = 0.56, ER) were found in previous research [79]. Given the high correlation between subscales (*r*(86) = 0.80, *p* < 0.001) and the recommendation of recent literature [80] we utilized the total score (α = 0.92), where higher scores indicate greater difficulty with emotion expression.

#### COVID-19-Related Posttraumatic Stress Symptoms in Children

Developed by the University of California at Los Angeles, the 11-item Brief COVID-19 Screen for Child/Adolescent PTSD (UCLA-BCSCA) questionnaire was adapted from the UCLA PTSD Reaction Index for *DSM-5* Brief Form (RI-5-BF) [81, 82]. Children reported their COVID-19-related PTSS (e.g., “When something reminds me of what happened or is still happening, I get very upset, afraid, or sad”) on a Likert scale ranging from 1 (*None of the time*) to 5 (*Most of the time*). Psychometric properties for this scale are unavailable due to its novelty, but the scale from which it was adapted had excellent internal consistency (α > 0.93) and discriminant validity (*d* = 2.48). Reliability for the total sum score in the current study was excellent (α = 0.92); higher scores correspond to more symptoms of COVID-19 related PTSS.

#### Demographics

Parents reported demographic information. Mass disaster literature suggests that child age, child gender, parent gender, child race, socioeconomic status, and child psychopathology such as anxiety and depression should be considered as control variables [1, 2, 14]. Concerning the study of alexithymia specifically, there is evidence to suggest controlling for depression given the strong association between conditions [83, 84]. Therefore, we tested the following parent-reported constructs as potential covariates: child age, child gender, parent gender, child race/ethnicity, family socioeconomic status (i.e., family income and parent education), and child psychopathology. The latter was assessed via four questions asking whether or not the child had an anxiety or depression diagnosis before or during the pandemic.

### Data Analysis

We conducted all analyses in SPSS (Version 28.0). We screened the data for: (1) missingness, (2) univariate normality, outliers, and linearity, (3) multivariate normality, outliers, linearity, and homoscedasticity, and (4) multicollinearity [85]. Data fit all necessary assumptions with no transformations needed. Next, we ran a series of correlations and *t*-tests to examine the child (age, gender, race, anxiety, and depression), parent (gender), and family (socioeconomic status) sociodemographic variables. None were significantly associated with outcome or mediator variables, and were not included in main models.

## Results

The summed TAS-20 scores ranged from 12 to 46 (*M* = 26.52, *SD* = 9.28). The TAS-20 score was significantly correlated with EESC (*r* = 0.42, *p* < 0.001) and PTSS (*r* = 0.64, *p* < 0.001) scores. The EESC and PTSS were also significantly correlated (*r* = 0.57, *p* < 0.001), and the EESC scores ranged from 16 to 65 (*M* = 40.66, *SD* = 12.62) while the PTSS scores ranged from 0 to 34 (*M* = 11.73, *SD* = 9.10). Table 1 provides the mean, standard deviation, reliability coefficients and correlations among all variables. About one-fifth (21.6%; *n* = 19) of children in this sample had high PTSS - scores above the cutoff for a PTSD diagnosis [81, 82] - while 29.5% (*n* = 26) had mild PTSD symptoms; the remaining 48.9% (*n* = 43) were only minimally symptomatic.

**Table 1:** Measure descriptives

				*r*	
	M	SD	α	EESC	PTSS
TAS	26.52	9.28	0.89	0.42*	0.64*
EESC	40.66	12.62	0.92	–	0.57*
PTSS	11.73	9.10	0.92	–	–

*Note* *Correlation significant at the 0.001 level (2-tailed)

**Fig. 2:**
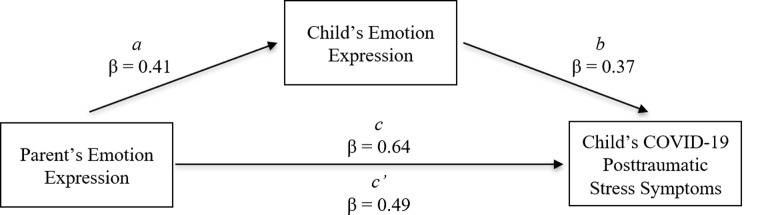
.

Using 5000 bootstraps with replacement, we examined the bias corrected 95% confidence intervals to determine if there were statistically significant direct and indirect paths from parent alexithymia to PTSS through child emotion expression using Model 4 in the PROCESS 4.0 macro [86] for SPSS. There was a statistically significant direct pathway from parent alexithymia to PTSS (β = 0.64, *SE* = 0.08, *p* < 0.001) as well as a statistically significant indirect pathway from parent alexithymia to PTSS through child emotion expression (β = 0.15, *SE* = 0.05, 95% CI: [0.07, 0.25]). The standardized coefficient, standard error, and significance value for each pathway, including the hypothesized direct path, can be found in Table 2. The path model is also visually represented in Fig. 2. Last, we tested the alternative moderator model to determine whether there was a significant interaction effect of parent alexithymia with child emotion expression on child PTSS. Predictor variables were mean centered prior to analysis and a product term created, then we ran a hierarchical linear regression to test the direct effects of parent emotion expression and child emotion expression on child COVID-19 PTSS [87]. The interaction effect was not significant (β = 0.06, *p* = 0.44).

**Table 2:** Indirect and total effects

						95% CI	
Effect Type	Path	*B*	*SE*	β	*p*	Lower	Upper
Component	a	0.56	0.13	0.41	< 0.001	0.30	0.83
	b	0.26	0.06	0.37	< 0.001	0.14	0.38
Direct	c’	0.48	0.08	0.49	< 0.001	0.31	0.64
Indirect	a-b	0.15	0.05	0.15		0.07	0.25
Total	c	0.62	0.08	0.64	< 0.001	0.46	0.79

*Note* Results hold even when controlling for depression

## Discussion

The current study considered child and parent factors that potentially influence heterogeneous child stress responses to COVID-19. Results supported the exploratory path model, wherein the direct effect was partially explained by greater child alexithymia symptoms; greater parent alexithymia symptoms were associated with poorer child emotional functioning, which in turn was associated with higher child COVID-19-related PTSS. The alternate moderator model was not supported.

Findings corroborate and expand upon existing mass trauma research by suggesting the intergenerational transmission of emotional processing difficulties and a link between greater emotion-related difficulties in parents and greater child PTSS. This may be because children witness and reflect their parents’ stress, though it seems more likely that this association is due to a lack of emotion processing. The current study explored a pathway through which this association between parent alexithymia and child COVID-19-related PTSS might be explained: the intergenerational transmission of alexithymia. Emotion socialization and parent meta-emotion philosophy have been noted as particularly important indicators of the attention parents devote to helping their children identify and understand emotions [88]. It is likely that parents’ meta-emotion philosophy, including their ability to understand and express their emotions, informs such socialization. Likewise, parents’ mentalizing and sensitivity in the attachment relationship may be implicated in the transmission of emotion. Parents with higher alexithymia symptoms may be less able to mentalize, respond sensitively, and devote attention to positive emotion socialization processes due to their own lack of emotional understanding and processing. It stands to reason that parents cannot teach what they do not understand. This pattern, where the socialization practices of parents with alexithymia have been directly implicated as problematic for children’s emotional development, has been evidenced in numerous studies [35–38]. Our findings expand upon this work by exploring the socialization pattern as a risk factor during mass trauma. We conclude that when parents are less likely to be able to guide and encourage children’s own understanding and processing of emotion and/or dismiss or minimize emotions, whether intentional or inadvertent, the outcome is the same: children do not learn to identify and express their own emotions and in turn struggle to process emotional events such as the COVID-19 pandemic.

Our findings are particularly important given the developmental stage of children in our sample. Middle childhood is a time of rapid development and when alexithymia symptoms begin to present [89]. During this developmental period, children refine their emotional understanding, expression, and regulation skills, making them particularly susceptible to the effects of impaired emotional socialization [90]. Additionally, parents serve as primary models for emotion regulation, with children observing how their parents handle emotions like stress and conflict, thereby learning coping mechanisms and emotional responses. Moreover, parents significantly influence the socialization of emotions. Active listening and empathetic responses from parents can foster emotional understanding and create a safe environment for children to express feelings. In such a secure environment, parents can serve as a safe haven for their children during stressful times and buffer the effects of traumatic life events, including the COVID-19 pandemic [91–93]. Our data provides preliminary evidence for the transmission of alexithymia from parent to child and suggests that this transmission carries consequences for children’s processing of traumatic events. Findings underscore that parents with high alexithymia symptoms may struggle to provide effective emotional socialization and support, potentially hindering their children’s emotional competence and ability to manage stressors. Future work would benefit from longitudinal investigations of the pathway between alexithymia symptoms and stress outcomes and investigations of how emotion-based interventions targeting the parent-child dyad could mitigate such outcomes. Our findings suggest the active role children can play in their experience. Although children’s emotion regulation is most prominently influenced by parents, other factors that may promote healthy development should be investigated.

The prevalence of PTS in this study was somewhat disparate from that obtained from other studies of individuals who experienced mass trauma. In those studies, PTSD prevalence rates ranged from 30 to 50%, even years after the event [5, 9, 94, 95]. While the current study analyses used a continuous PTSS scale, descriptive diagnostic categories revealed that only one in five children had a potential PTSD diagnosis; just under a third of the sample was classified with mild PTSS and nearly half of the sample was only minimally symptomatic. Our lower prevalence rates may be due to the time lapse between when COVID-19 was more immediately and acutely affecting families (early 2020) versus when data was collected (2021). Even still, the fact that over one in five children experienced PTSS that would potentially translate to a PTSD diagnosis underscores the traumatic effect that COVID-19 has on children.

Our data supported child emotional competence as a mediator, but not a moderator, of the relation between parental emotion competence and child PTSS. The lack of support for a moderator model further validates our key study finding, providing additional evidence that alexithymia is intergenerationally transmitted rather than operating as a synergistic influence on stress outcomes. One implication of the fact that child alexithymia mediated the association between parent alexithymia and child PTSS is that involving parents in prevention and intervention work intending to mitigate child internalizing psychopathology is critical, as opposed to focusing exclusively on increasing children’s individual emotion regulation skills. Improving emotion-based communication between parent-child dyads should be explored in more depth. Our findings indicate some level of dependency between parent and child alexithymia; while the child remains an active agent, parent emotion socialization practices are particularly influential on child outcomes. In the context of COVID-19, families were unexpectedly isolated from other social supports [96]. Child inequities in the ability to emotionally process the mass trauma of COVID-19 were thus partially dependent on the emotional competence of parents. Future work should investigate this further, such as exploring how emotional climates shifted during COVID-19 and how these associations may operate cyclically.

### Limitations and Future Directions

This study had several limitations. First, the data were cross-sectional. Ideally, mediation requires temporal order of the measured variables [87]; without this, the assumption of uncorrelated errors that underlies mediation analyses is suspect. However, mediation with cross-sectional data should not be ruled out entirely, as cross-sectional analyses grounded in theory that describe causal direction of the measured processes should be considered [97]. Given the theoretical bases for the current model, existing literature on the associations between alexithymia and PTSS, and the lack of support for the alternative model, we argue that the current findings are valid and valuable. Regardless, these findings are best considered exploratory and suggestive as opposed to conclusive until they are replicated with longitudinal data. Findings should be corroborated with longitudinal work that can establish directionality, and future research could test theorized mechanisms of transmission (e.g., direct teaching/socialization, caregiver sensitivity, mentalizing, and emotional unavailability).

Additionally, parent-child dyads were not analyzed as paired units, but instead the continuous scores of each construct were utilized. While results provide valuable insight into the associations between parent-child alexithymia scores, it would be advantageous to explore these associations within the dyadic unit. Future work should corroborate these findings through repeated measures and other longitudinal designs and consider examining factors such as dyadic cohesion, attachment behaviors, or communication for further insight into the processes informing the parent-child emotional climate.It would also be interesting to focus on the child’s contribution. For example, an emotional availability perspective would incorporate the child’s contribution to emotional regulation in the dyadic relationship (e.g., by directly examining the child’s responses during parent-child interactions) [98].

Furthermore, other variables not included in the current study could have affected the associations between the key study constructs. For example, geographic location and familial exposure or death due to COVID-19 [2, 4, 14] might have influenced our results. Interestingly, none of the available covariates were associated with children’s emotional competence or PTSS. Future work should seek to replicate this finding with a larger sample and explore why variables which are usually correlated were unrelated under alexithymia and COVID-19 conditions.

Lastly, recruitment challenges and time constraints resulted in a modest sample size and somewhat limited racial/ethnic and economic diversity. Though measurement errors were generally low, and the study retained adequate power to detect medium effects, it would be beneficial for future work to expand the sample to further validate these findings. Generalizability of the current findings is limited, as the sample fits the WEIRD (Western, Educated, Industrialized, Rich, Democratic) [99] classification. Future work should corroborate these findings in more diverse samples. It would also be interesting to examine how culture or associated values and experiences influence the proposed model. For example, a full understanding of how African American children develop emotionally requires consideration of mainstream and minority cultural influences (e.g., Afrocultural ethos) [100].

## Conclusions

Findings highlight the importance of emotion processing and regulation during mass trauma, particularly how alexithymia is transmitted from parent to child and subsequently informs child adjustment. To our knowledge, this is the first study to evidence this effect with specific regard to COVID-19-related outcomes. Individual emotional health and the parent-child emotional climate can be areas of focus for psychoeducation and intervention during mass traumas such as pandemics.

### Summary

Children with PTSS can face substantial impairment in social functioning, academic performance, and subsequent mental health; therefore, exploring factors which contribute to the parent-child emotional climate, as well as the subsequent psychological effects, is critical to understanding how heterogeneous stress outcomes develop among youth, particularly in the context of COVID-19. The current study utilized emotion socialization and meta-emotion philosophy to orient the hypothesized intergenerational transmission of alexithymia (i.e., a difficulty identifying and expressing emotion), from parent to child, as it informs children’s COVID-19-related PTSS. As predicted, parents’ emotional expression challenges (i.e., greater difficulty identifying feelings and difficulty describing feelings) were positively associated with their children’s COVID-19-related PTSS. Further, the association between parent alexithymia and child PTSS was partially explained by children’s emotional expression abilities, such that parents’ greater difficulty identifying and describing feelings were associated with poorer emotional awareness and more expressive reluctance in children, which in turn was associated with greater child PTSS. In contrast, the alternative moderator model was not supported. Findings corroborate and expand upon extant work, suggesting that emotional processing difficulties can transmit from one generation to the next, and ultimately inform children’s mental health outcomes. Thus, parental involvement in preventive intervention work is critical in order to expand on their knowledge of emotions and mitigate consequences of intergenerationally transmitting difficulty with emotions. More specifically, there is a need to develop and test interventions across potential pathways of this transmission, such as parents’ sensitivity to emotional cues, as well as emotional understanding (e.g., mentalization), availability, and socialization in order to reduce children’s negative mental health outcomes. Future work should examine factors that may promote healthy development and utilize longitudinal methods in order to develop robust interventions.

## Data Availability

This study was not pre-registered. The data that support the findings of this study are available from the corresponding author upon reasonable request.

## Declarations

### Permission to Reproduce Material from Other Sources

Authors did not reproduce material from other sources.
